# Rabbit VX2 head and neck squamous cell models for translational head and neck theranostic technology development

**DOI:** 10.1002/ctm2.550

**Published:** 2021-10-12

**Authors:** Nidal Muhanna, Catriona M. Douglas, Harley H.L. Chan, Michael J. Daly, Jason L. Townson, Marco Ferrari, Donovan Eu, Margarete Akens, Juan Chen, Gang Zheng, Jonathan C. Irish

**Affiliations:** ^1^ Guided Therapeutics (GTx) Program Princess Margaret Cancer Centre/University Health Network TECHNA Institute Toronto Ontario Canada; ^2^ Department of Otolaryngology‐Head and Neck Surgery‐Surgical Oncology Princess Margaret Cancer Centre/University Health Network University of Toronto Toronto Ontario Canada; ^3^ Department of Otolaryngology–Head and Neck Surgery Tel Aviv Sourasky Medical Centre Tel Aviv University Tel Aviv Israel; ^4^ Unit of Otorhinolaryngology—Head and Neck Surgery University of Brescia Brescia Italy; ^5^ Department of Medical Biophysics University of Toronto Toronto Ontario Canada; ^6^ Institute of Biomaterials and Biomedical Engineering University of Toronto Toronto Canada

Dear Editor

The study aims to provide an overview of the key aspects of the VX2 rabbit head and neck model and how it can be used in translational research, with a particular focus on theranostic technology development.

Our extensive experience in preclinical multimodality imaging and phototherapy technology to guide surgery has enabled us to identify key features that are required in an animal model to perform Head and Neck translational research: physical size large enough to allow tumors to be visible on microimaging modalities, a model that allows application of phototherapy instrumentation and fluorescence guidance, an immunocompetent model, and ability to grow tumor at different head and neck subsites, see Figure 1. Furthermore, an animal model that allows survival surgical treatment to be performed is advantageous when assessing new treatments.

**FIGURE 1:**
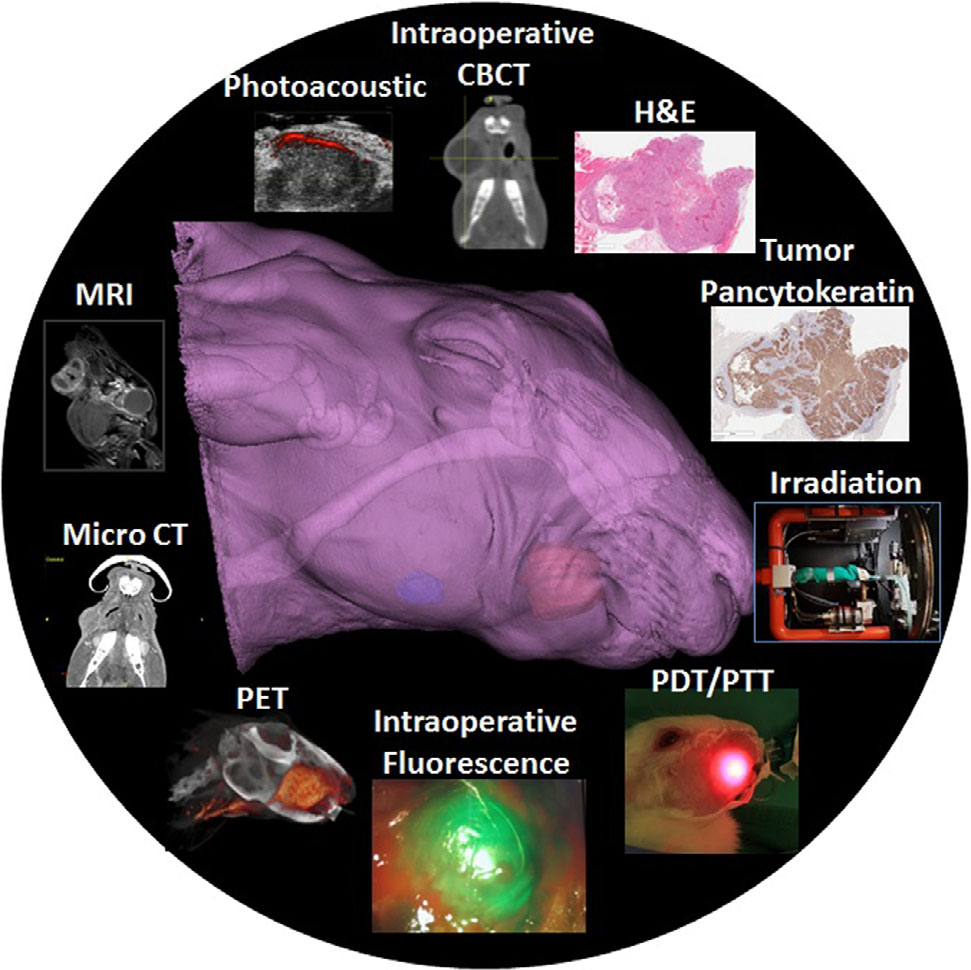
Overall imaging modalities that can be used in the Rabbit VX2 Head and Neck Cancer model for diagnostic, disease monitoring, and image‐guided procedures: intraoperative cone‐beam CT, photoacoustic, MRI, PET, intraoperative fluorescence, and microCT

The rabbit VX2 model is a long‐established model in head and neck cancer, first reported in 1936 by Kidd et al.^1^ The VX2 tumor is derived from the cottontail rabbit papilloma virus, which, like the human papilloma virus (HPV), participates in papillomatosis and carcinogenesis of squamous epithelium.^2^ Head and neck tumors were generated by injection of 100 microliters of VX2 cell clusters from tumor tissue (propagated in the thigh of another rabbit) into either anterior tip of the tongue or buccinator muscle. All animals in this study were anesthetized for surgery using the inhalant anesthetic isofluorane, see Supporting Information. The methodology that we employed in the VX2 Head and Neck model has a 100% tumor uptake rate in primary tumors developed, and commonly lymph node metastasis, within a matter of 2‐3 weeks, see Figure 2.

**FIGURE 2:**
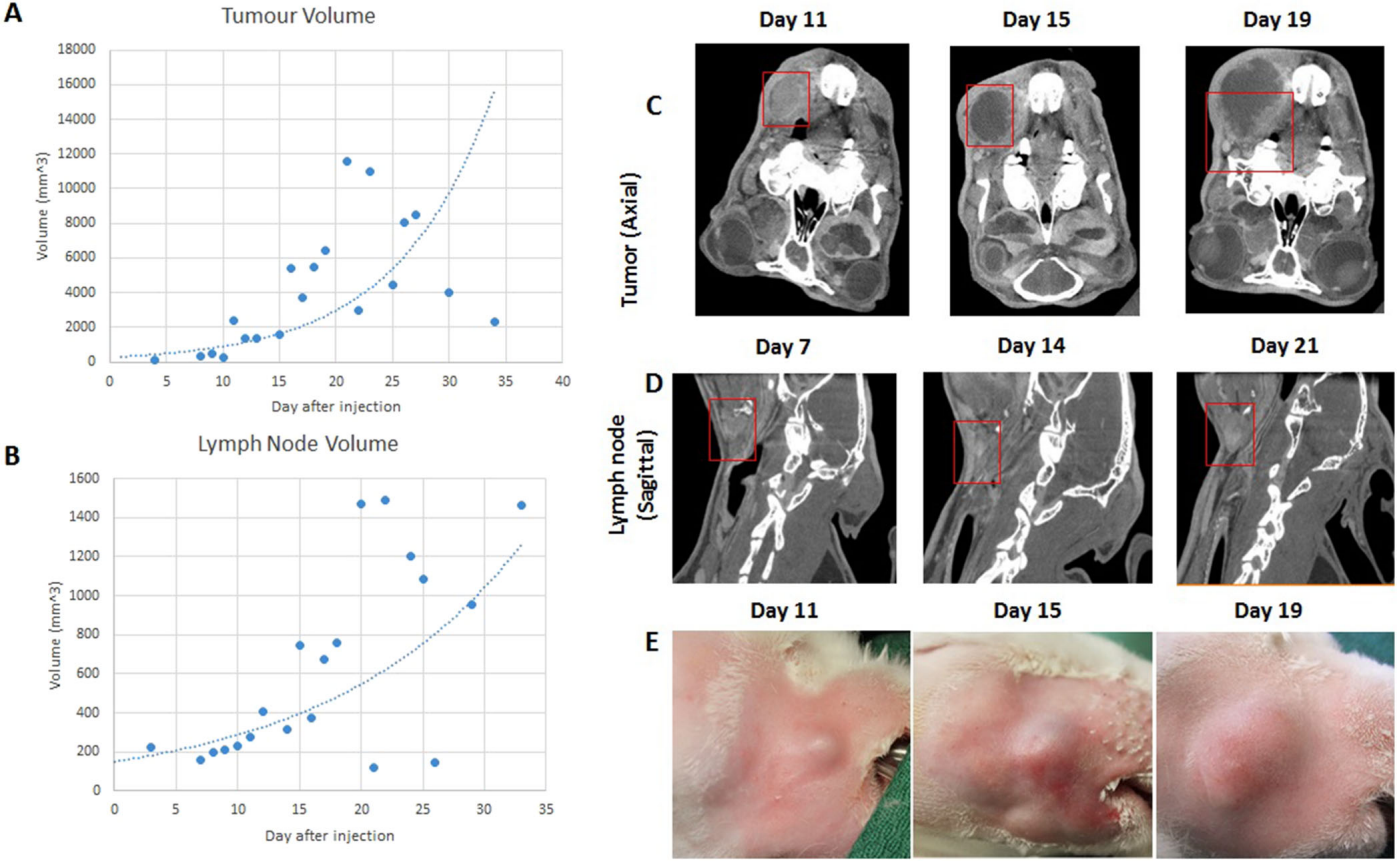
Buccal tumor characteristics in the rabbit VX2 model. (A) Tumor volume mm^3^ assessed by microCT plotted against time after injection. (B) Cervical lymph node metastasis volume mm^3^ assessed by microCT plotted against time after injection. (C) Axial microCT images of buccal tumor at day 11, 15, and 19. (D) Sagittal microCT images of lymph node metastasis at day 7, 14, and 21. (E) Clinical photograph of VX2 buccal tumor at 11, 15, and 19 days

A total of 430 rabbits were used for these studies, the most common tumor site was buccal region (74%), followed by tongue. Rabbits were imaged using several different modalities preoperatively and intraoperatively, see Figure 3, Supporting information Table S1. The treatments rabbits received were photodynamic therapy (PDT), photothermal therapy (PTT), external beam radiotherapy and survival surgery with excision of the tumor and cervical lymph nodes, see Figure 4.

**FIGURE 3:**
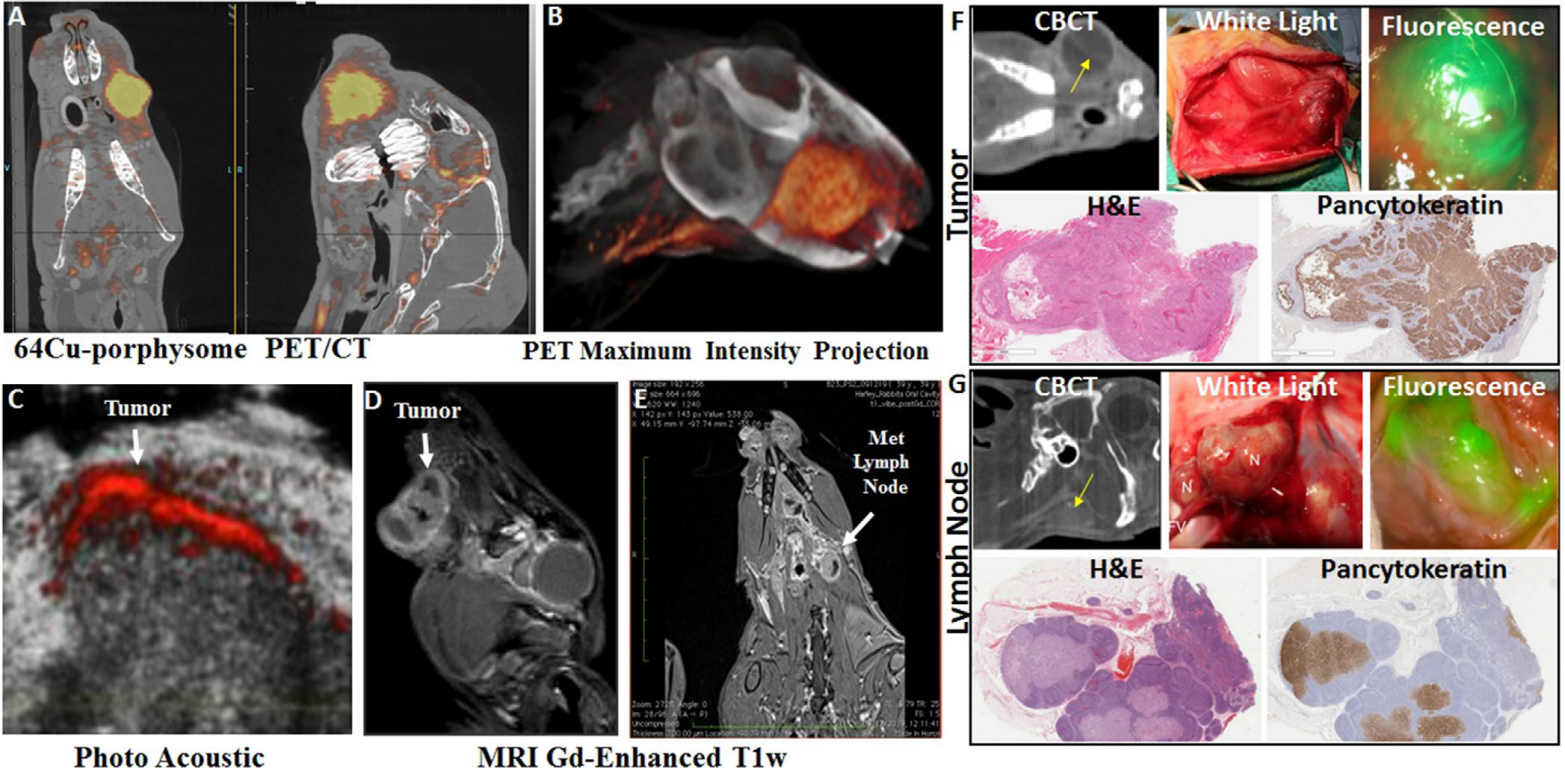
Preoperative imaging of VX2 buccal tumor: (A) coronal, sagittal, and axial 64Cu‐porphysome PET/CT imaging. (B) Maximum projection intensity overlay with PET signal. (C) Photoacoustic imaging. (D,E) T1‐weighted gadolinium‐enhanced MRI of tumor (D) and metastatic cervical lymph node (E). (F) Intraoperative imaging and postoperative histology of buccal cancer with metastasis model

**FIGURE 4:**
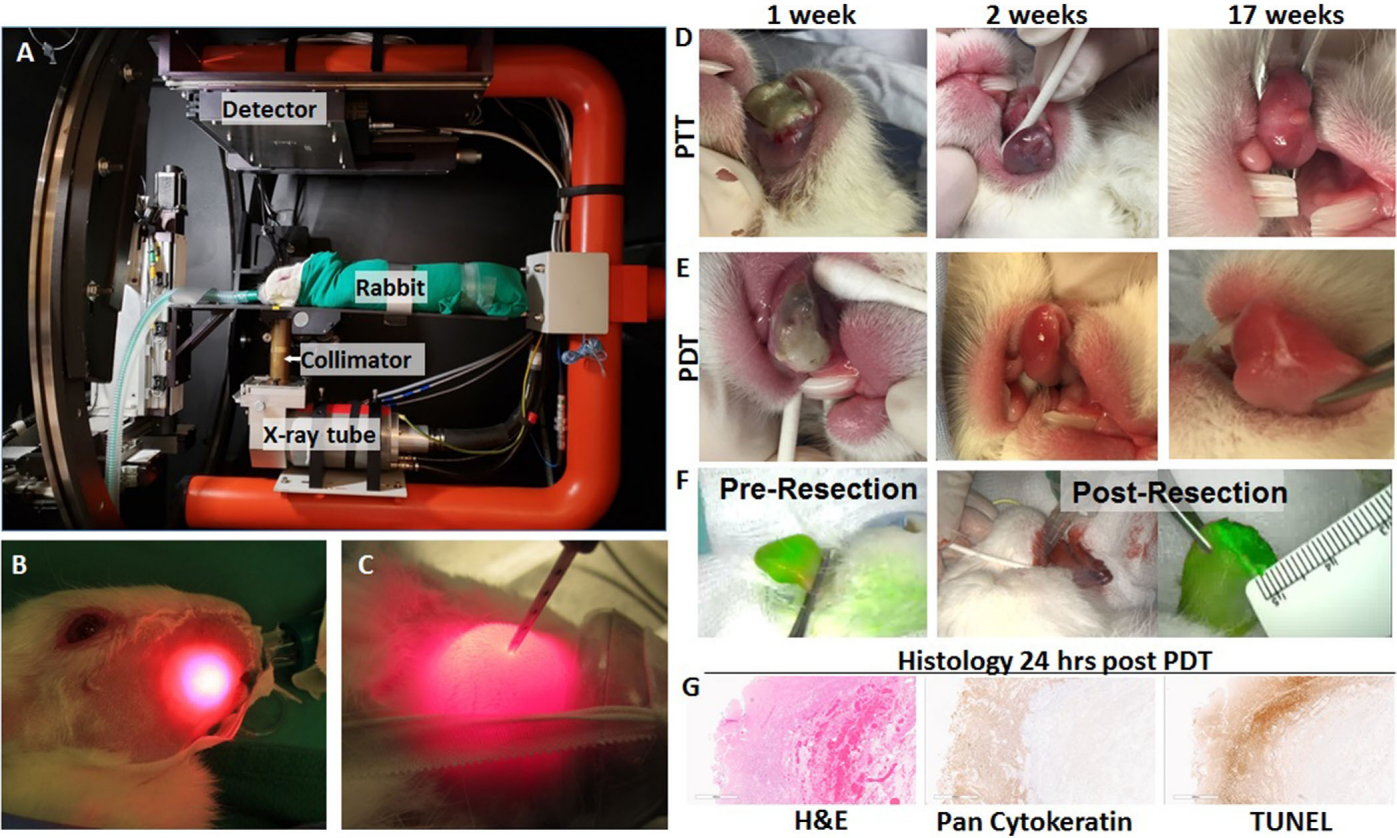
Treatment of buccal VX2 tumors. (A) Micro‐IGRT (image‐guided radiation therapy) unit for small‐animal radiotherapy. (B) Intralesional photodynamic therapy. (C) 671 nm laser light for photodynamic therapy. (D) 1 week, 2 weeks, and 17 weeks after treatment with photothermal therapy (PTT). (E) 1 week, 2 weeks, and 17 weeks after treatment with photodynamic therapy (PDT). (F) Pre‐ and Postresection after nanoparticle injection. (G) H&E, pancytokeratin and tunnel staining of rabbit tongue 24 h after photodynamic treatment

This model has cervical lymph node anatomy similar to humans, making it a useful model for assessing cervical metastasis. Clinical nodal metastasis was evident in 80% of the rabbits. The anatomical size of the rabbit allowed for survival surgical procedures to be performed on the oral cavity tumor and buccal tumor, with intraoperative assessment of the tumor with cone‐beam CT (CBCT), and the use of fluorescence to guide surgery, see Figure 3.^3^, ^4^ Radiotherapy, using planning software, PDT and PTT can all be delivered to these tumors, see Figure 4.

As a preclinical animal model for HPV‐related HNSCC circulating tumor DNA (ctDNA) release into the circulation from tumor site and plasma HPV DNA kinetics was evaluated.^11^ To detect ctDNA derived from tumor cells, a qPCR assay for use on cell‐free plasma DNA was used (Supporting information Figure S1). The role of E6 oncoprotein is well established in HPV positive oropharyngeal infection. In the VX2 rabbit model, the E6 oncoprotein is amplified within VX2 genomic DNA, making this a highly relevant biological model for use in head and neck research. Circulating tumor cells were captured based on EpCAM expression, see Supporting information. This model is there for both biologically relevant to HPV‐driven HNSCC and also anatomically relevant to HNSCC.

The growth of the tumor was consistently reliable, allowing for clinical examination under anesthetic and cross‐sectional imaging to be performed weekly. Surgery can normally be planned within 2 weeks of VX2 cell inoculation. This reliable growth pattern allows for experiments to be planned in a timely manner, in comparison to some other animal models. Subcutaneous xenograft mouse models are often used in preclinical studies; however it is often noted that these models lack the specific microenvironment interactions between the tumor and normal host tissue. A further disadvantage of using xenograft models with human cell lines is that it requires the use of immunocompromised mice, precluding the study of any interactions between the host immune system and the tumor. The small physical scale of the mouse makes any head and neck orthotopic model technically demanding, and any surgical procedure very difficult to perform, see Supporting information Table S2.

The size of rabbits allows for the use of a wide variety of clinical imaging systems, facilitating longitudinal and noninvasive imaging to reduce the number of animals required for each study. Serial blood drawing constitutes an important aspect of cancer research studies facilitating PK/PD (pharmacokinetics/pharmacodynamics) modeling and toxicity experiments. This is often difficult in smaller animals, such as mice, due to the very small circulating volume. An advantage of the rabbit head and neck model is that the marginal vein of the ear can be cannulated, allowing for a series of injections with anesthesia, drugs and fluids, while also allowing for repetitive blood samples to be taken (see Supporting information Figure S2 and supplementary text).

The risk of anesthetic‐related death in rabbits is very low (1.39% in healthy animals).^5^ Sick animals have a substantially higher risk of anesthetic‐related death, highlighting a population in which extreme care is required in the perioperative and postoperative period. The postoperative monitoring of the rabbit after any procedure is critical to ensure the rabbit recovers well from any intervention. We have a very rigorous postoperative care plan as agreed in our animal use protocol including pain medications, fluids, nasal gastric tube, and antibiotics (Supporting information).

This HNSCC preclinical model has been used extensively by our group investigating lipid‐based nanoparticle for preoperative imaging (CT, PET/CT)^3^, ^6^ and intraoperative imaging (CBCT, fluorescence)^9^ for both PDT and PTT treatment of buccal SCC. Based on the experience of this anatomical model and knowledge, we are moving toward a first‐in‐human theranostic clinical study. This animal model has proved to have several advantageous properties that have aided in the development and investigation of alternative imaging and therapeutic approaches for head and neck cancer therapy.

The biological behavior of this animal model closely resembles that of human head and neck squamous cell carcinoma. Biologically, it also closely resembles HPV‐mediated HNSCC. The consistent growth of the tumor, along with the high rates of cervical metastasis makes it a useful model for assessing treatment regimens targeting both the primary and nodal metastasis.

## DATA AVAILABILITY STATEMENT

Data available on request from the authors.

The data that support the findings of this study are available from the corresponding author upon reasonable request.

## Supporting information

Supporting Information

Supporting Information

Supporting Information
