# Supplementary material for: Rabbit VX2 head and neck squamous cell models for translational head and neck theranostic technology development
Source: Clin Transl Med. 2021 Oct 12;11(10):e550. doi: 10.1002/ctm2.550 (PMC8506636; doi:10.1002/ctm2.550)
Supplement: Supplementary file 1 — Supporting Information [file CTM2-11-e550-s001.docx]

Supplemental Work for VX2.

All animal studies in this letter were performed according to Animal Usage Protocols (AUP) approved by an ethical review board for animal research at our institute (University Health Network – University of Toronto).

**Table 1. Imaging modalities in the rabbit head and neck model.**

| **Imaging Modality** | **Advantages of modality in Rabbit model.** | **Routinely used in Assessment of Human Head and Neck Cancer** | **Manuscript Figure** | **References** |
| --- | --- | --- | --- | --- |
| microCT | Quick. Short Anaesthetic. High-resolution images. | Yes | 4, 8 | ^6,7^ |
| Cone beam computed tomography | Very quick to capture. Can be used intraoperatively. | No | 6 | ^8^ |
| *Magnetic resonance imaging* | Soft-tissue resolution. | Yes | 5, 8 | ^9^ |
| *PET imaging* | Whole body imaging. Contrast agent. | Yes | 5 | ^10^ |
| *Photoacoustic* | Anatomical size enables photoacoustic imaging due to probe size. Able to see 3D view of tumor. | No | 5 | ^10^ |
| *Intraoperative fluorescence imaging* | Localisation of tumor and lymph node metastasis. | No | 6 | ^9^ |

Table 2. Animal head and neck models pros and cons

| Animal Model | Pros | Cons |
| --- | --- | --- |
| Rabbit | Large size. Serial blood drawing. longitudinal survival studies. Safe to give multiple anaesthetics.  Immune competent. | Expensive animal husbandry.  VX2 tumor not well characterised. |
| Hamster | Moderate size.  Tumor starting at mucosal surface and infiltrating.  Immune competent.  Mixed phenotype (dysplasia to tumor). | Very long time (months) to generate tumor.  Regular application of carcinogen required to develop tumor.  Multiple tumor nodules, not discrete lesion.  Low rate of lymph node and distant metastasis. |
| Mouse | Human cell lines used for tumor.  Large numbers can be used.  No consistent nodal models. | Immunocompromised.  Unable to perform serial blood drawing. |
| Cat | Large size. | Naturally occurring tumor. Poor outcome. Highly invasive tumor. |

Circulating Tumour Cell assessment.

Moreover, CTCs were selectively captured based on EpCAM expression using immunomagnetic nanoparticles depends on antibodies against EpCAM attached to magnetic nanoparticles allowing for capture using a magnetic field^12^. The number of CTCs was correlated to the tumor volume and decreased immediately after tumor resection.

Detection and quantification of CRPV DNA from the VX2 tumor cells has been demonstrated in a previous publication by the lab^11^.

*VX2 carcinoma rabbit model in the head and neck.*

The VX2 squamous cell carcinoma models were developed using the method described by Rous et al[4, 5]. The VX2 tumor is a squamous cell carcinoma characterized by rapid growth, hypervascularity, and facile propagation in skeletal muscle. Tumor was harvested under sterile conditions from the freshly euthanized rabbit used for tumor propagation, placed in HBSS, washed twice with sterile HBSS, cut into small pieces, and stored at -80°C until used. To obtain a tumor cell suspension, the tumor pieces were thawed, minced and pressed through a 70 µm cell strainer. Three hundred microliters of a high-density single-cell suspension (5 × 10^6^ cells/mL) was injected into the buccinator muscle (buccal area) of an anaesthetized rabbit using 27G needle. Tongue tumors were generated by injection of 100 microliters of VX2 cells into the anterior tip of the tongue.

*Anesthesia*

All animals in this study were anesthetized according to our local AUP protocol. Typically, the rabbit was given the inhalant anaesthetic isofluorane, and a rabbit laryngeal mask airway (LMA) was used to provide an airway, see figure 1. Five percent isofluorane was used for anesthetic induction with a small mask and maintenance at 2.5% with air flow at 2 L/min. Vital signs were monitored by oximeter including saturated O2 (typical value at 97~99%), heart rate (typical value at ~237bpm) and end-tidal CO2 monitoring (typical value ~30mmHg) while the rabbit was under anaesthesia, see figure 1. The rabbit LMA allows for good access to the oral cavity to perform surgery. In cases that surgery was not performed, 2.5% air flow at 2 L/min through the face mask was sufficient.

*Blood Collection*

Routine blood haematology and biochemistry can be performed on rabbit blood, allowing monitoring of progression of disease^5^. The ability to have the animal survive repeated blood drawing can help reduce the number of animals required as they can serve as their own control, unlike mice. Here, the marginal vein of the rabbit ear was used for blood collection. The site was prepared by wiping the area with a 95% alcohol swab. Only visible ear vessels were used. A 22G, 0.9 x 25mm angiocath (BD Angiocath^TM^) with needle bevel superior was inserted parallel into the marginal vein. A flashback of blood in the angiocath confirmed the cannula was in the correct position. Blood samples were taken at this point. A non – DEHP catheter extension set (Baxter) then placed on the catheter, allowing serial injection of anesthesia, drugs and saline. The canula was secured with an ear bolster (see figure 1).

*Nasogastric tube (NG) insertion*

A nasogastric tube may be inserted during the early post-treatment period to allow for continual nutritional maintenance following surgeries of the oral cavity. This may be performed with the rabbit awake or under general anesthesia. In the awake technique, the rabbit is restrained and topical anesthesia (proparacaine hydrochloride 0.5%) placed to the nasal cavity. A size 6 Fr 55 cm NG tube is then measured from the nasal cavity to the animal’s abdomen and the length recorded. The NG tube is then lubricated with aqueous jelly and inserted into the nasal passage to the desired length. The tube is withdrawn if the rabbit coughs or chokes during insertion. Following insertion, a test bolus of 3mL of water is administered and if coughing is observed the NG tube is withdrawn and procedure repeated. When the tube is placed accurately, there should be no reaction following the test bolus. The tube is then anchored to the snout, head and back following administration of local anesthetic. A collar is then placed over the rabbit’s neck to avoid dislodgement of the NG tube.

The NG tube may also be placed under general anesthesia. When the rabbit is under anesthesia, a size 6 Fr 55 cm NG tube is lubricated and inserted via the nasal passage. A portable X-ray is then used to visualize the distal tip of the NG tube to ensure it is below the level of the diaphragm. Following this, the tube is anchored and a collar placed over the neck as previously described (figure 1).
