# Supplementary figures and images for: Rabbit VX2 head and neck squamous cell models for translational head and neck theranostic technology development
Source: Clin Transl Med. 2021 Oct 12;11(10):e550. doi: 10.1002/ctm2.550 (PMC8506636; doi:10.1002/ctm2.550)

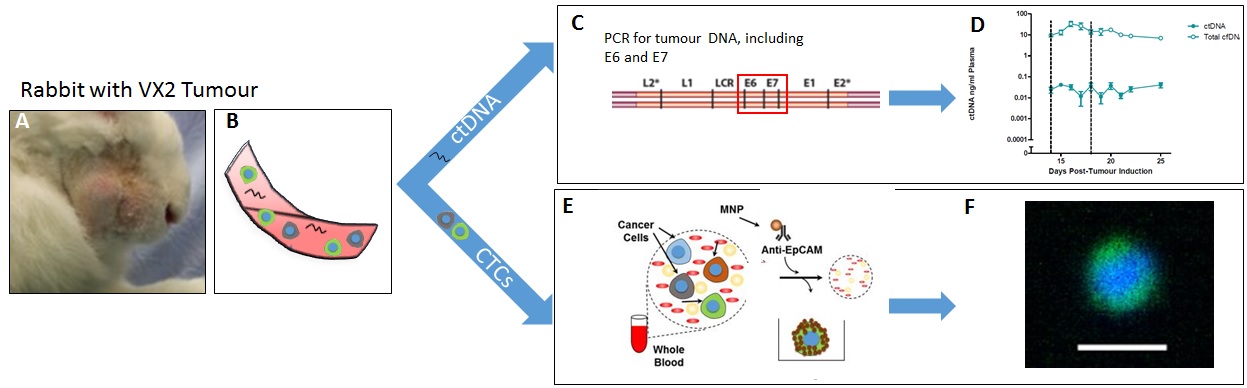

Supplement: Supplementary file 2 — Supporting Information [file CTM2-11-e550-s003.jpg]

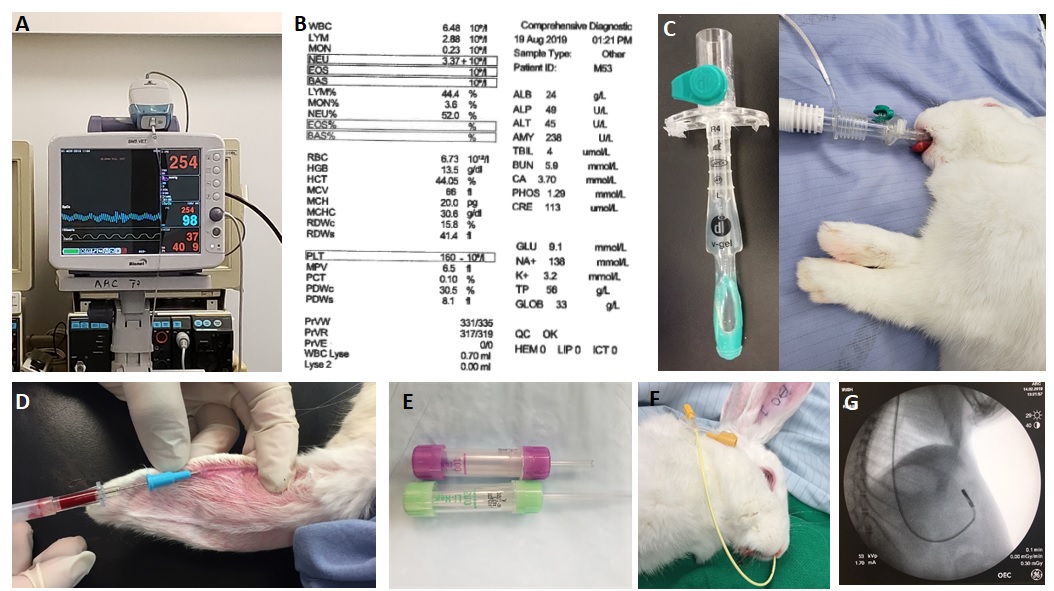

Supplement: Supplementary file 3 — Supporting Information [file CTM2-11-e550-s002.jpg]
